# Immunocastration in adult boars as a model for late‐onset hypogonadism

**DOI:** 10.1111/andr.13219

**Published:** 2022-07-08

**Authors:** Nina Batorek‐Lukač, Kevin Kress, Marjeta Čandek‐Potokar, Gregor Fazarinc, Martin Škrlep, Klavdija Poklukar, Raffael Wesoly, Volker Stefanski, Milka Vrecl

**Affiliations:** ^1^ Animal Science Department Agricultural Institute of Slovenia Ljubljana Slovenia; ^2^ Behavioral Physiology of Livestock Institute of Animal Science University of Hohenheim Stuttgart Germany; ^3^ German Genetic Schweinezuchtverband Baden‐Württemberg e.V. Stuttgart Germany; ^4^ Department of Animal Science Faculty of Agriculture and Life Sciences University of Maribor Hoče Slovenia; ^5^ Institute of Preclinical Sciences Veterinary Faculty University of Ljubljana Ljubljana Slovenia

**Keywords:** anti‐GnRH, morphometry, pig, response to immunocastration, testicular mRNA expression

## Abstract

**Background:**

While immunocastration has been studied in male pre‐pubertal pigs, data on older, sexually mature animals are limited. To understand the physiological effects of androgen deprivation in the late sexual development phase, we compared mature immunocastrated boars (*n* = 19; average age = 480 days) to young male immunocastrated pigs (*n* = 6; average age = 183 days) and young entire males (*n* = 6; average age = 186 days) as positive and negative controls, respectively.

**Objectives:**

We hypothesized that the timing of gonadotropin‐releasing hormone suppression (early or late sexual development phases) influences the extent of reproductive function inhibition, histological structure of testicular tissue, and expression levels of selected genes related to steroid metabolism.

**Materials and methods:**

Antibody titer, hormonal status, and histomorphometric analysis of testicular tissue were subjected to principal component analysis followed by hierarchical clustering to evaluate the immunocastration effectiveness in mature boars.

**Results:**

Hierarchical clustering differentiated mature immunocastrated boars clustered with young immunocastrated pigs from those clustered with entire males. Although all mature immunocastrated boars responded to vaccination, as evidenced by the increased gonadotropin‐releasing hormone antibody titers (*p* < 0.001), decreased serum luteinizing hormone concentrations (*p* = 0.002), and changes in testicular tissue vascularization (lighter and less red testicular parenchyma; *p *≤ 0.001), the responses were variable. Sharp decreases in testes index (*p* < 0.001), Leydig cell volume density (*p* < 0.001), Leydig cell nucleus‐to‐cytoplasm ratio (*p* < 0.001), and testosterone concentration (*p* < 0.001) were observed in mature immunocastrated boars clustered with young immunocastrated pigs compared with those that clustered with entire males. Additionally, mature immunocastrated boars clustered with young immunocastrated pigs showed lower hydroxysteroid 17‐beta dehydrogenase 7 expression than entire males (*p* < 0.05). The young immunocastrated pigs group showed higher follicle‐stimulating hormone receptors than the entire males and mature immunocastrated boars, lower steroidogenic acute regulatory protein expression levels compared with entire males, and mature immunocastrated boars clustered with entire males (*p* < 0.01).

**Conclusion:**

The two‐dose vaccination regime resulted in progressive but variable regression of testicular function in adult (post‐pubertal) pigs; however, it was insufficient to induce a complete immunocastration response in all animals.

## 1 INTRODUCTION

The regulation of mammalian reproduction requires the integration and precise orchestration of hormonal regulation of the hypothalamic–pituitary–gonadal (HPG) axis. Neurons of the periventricular and medial preoptic areas of the hypothalamus secrete gonadotropin‐releasing hormone I (GnRH‐I) in a pulsatile manner into the hypophyseal portal circulation to be transported to the anterior pituitary gland, where it interacts with the specific high‐affinity type I GnRH receptor (GnRHR‐I), promoting the synthesis and secretion of gonadotropins, luteinizing hormone (LH), and follicle‐stimulating hormone (FSH).^1^, ^2^ The release of LH and FSH is controlled by the magnitude and frequency of GnRH pulses through androgen and estrogen feedback^3^ as well as by direct inhibition of GnRH synthesis and release by gonadotropin‐inhibitory hormone, forming a dual control system of the pituitary gland gonadotropes.^4^ In males, LH controls the production of steroids (androgens, estrogens, and androstenes) via Leydig cells, whereas FSH is responsible for spermatogenesis in the seminiferous tubules.^5^ In addition to GnRH‐I, most vertebrates also express GnRH‐II, which is expressed predominantly in peripheral tissues; however, only a few mammalian species have a functional type II GnRH receptor (GnRHR‐II).^6^ Pigs are unique among livestock species because they have the largest testicular interstitial compartment, with the highest abundance of Leydig cells, suggesting a high capacity for steroid production.^7^ In boars, GnRH‐II and its receptor function as an autocrine/paracrine regulator of steroidogenesis within the testis.^8^ This is supported by (i) the predominant expression of GnRHR‐II in porcine Leydig cells, whose activation leads to LH‐independent secretion of testosterone^9^; (ii) observations that immunization against GnRH‐II reduces testosterone but not gonadotropin secretion^10^; and (iii) reports that, in transgenic swine with ubiquitous GnRHR‐II knockdown LH secretion is unaffected.^8^

In humans, testosterone deficiency, or male hypogonadism, is caused by intrinsic testicular failure (primary or hypergonadotropic) or suboptimal stimulation by pituitary gonadotropins (secondary, central, or hypogonadotropic). Both situations can be congenital, or acquired because of aging, disease, or androgen deprivation therapy. In male pigs, gonadectomy is required to prevent the accumulation of boar taint compounds (e.g., androstenone and skatole) in adipose tissue,^11^ which makes pork undesirable for sensitive consumers,^12^ as well as to prevent aggressive and sexual behavior in group housing.^13^ Gonadectomy is mainly performed without anesthesia or analgesia within the first 7 days of life.^14^ Alternatively, hypogonadism in male pigs can be achieved via the androgen deprivation method using vaccination against GnRH, also termed immunocastration (IC)/vaccination against boar taint, which has been available in the EU since 2009. The main purpose of IC in pig production is to reduce androstenone and skatole concentrations in fat tissue below the threshold level for the risk of boar taint (0.5–1 and 0.25 ppm, respectively^11^, ^12^). To achieve this, two vaccinations are required to trigger the production of anti‐GnRH antibodies that block the HPG axis, resulting in the suppression of testicular growth and function 14–28 days after revaccination in young boars.^15^, ^16^, ^17^ Although the vaccine can be used in boars of any age that are not intended for breeding, most data on IC were gathered using effective immunization in the early stages of pubertal development in male pigs slaughtered at standard slaughter weight and age (i.e., up to 120 kg live weight and 200 days of age^18^). In contrast, there is limited information on the use of IC at an advanced stage of sexual development: that is, older mature boars excluded from further breeding or at the end of their working life, which can extend to about 3–4 years of age. Although the number of such animals is quite low, IC could valorize meat from adult boars and increase their market value. Additionally, adult boars may be a suitable animal model for late‐onset hypogonadism, which occurs, for example, in patients receiving therapy for sex steroid‐dependent tumors.^19^ This is equivalent to pigs been suggested as a suitable animal model for translational research because of the similarities in their reproductive processes with those of humans.^20^ Evidence also supports the use of IC boars as a model for the study of secondary hypogonadism in humans.^21^, ^22^, ^23^ Regression of the reproductive tract in immunocastrated male pubertal pigs is consistent with a loss of functional activity, as shown by the histological changes in testicular tissue.^24^, ^25^, ^26^ Research findings suggest that the timing of vaccination (during either early or late sexual development stage) may play a crucial role in the extent of suppression of reproductive function^25^; however, the underlying physiological mechanisms are unclear and merit further investigation. As the transcript level of GnRHR‐II in the testis increase significantly only after puberty,^27^ its expression may be influenced by the timing of HPG axis suppression by IC. Therefore, the main objective of this study was to provide additional insight into the physiological effects of androgen deprivation in the late sexual development phase (adult boar model). Thus, we investigated the effects on reproductive function, including hormonal changes and antibody titers, histomorphology of testicular tissue, and testicular mRNA expression of selected genes associated with testicular function and steroid metabolism in mature immunocastrated boars (MICs) compared with those in young immunocastrated (YICs) and entire (uncastrated) male pigs (EMs) of standard fattening age (app. 185 days; positive and negative controls), to evaluate the response to IC in mature animals and determine which traits could be used to confirm the efficacy of IC.

## 2 METHODS

### 2.1 Animals and study design

This study consisted of two experiments. The control experiment was approved by the Ethical Committee for Animal Experiments at the regional level by the authority of Tübingen, Germany (permission for animal experimentation ID HOH 47/17TH). Boars in the main experiment were tested during routine diagnostics conducted by a veterinarian from the Besamungsstation Schwein in Herzberg. According to Directive 2010/63/EU (2010), the study was not subject to ethical protocols. The Veterinary faculty of the University of Ljubljana and the Agricultural Institute of Slovenia are also approved by the Veterinary Administration of the Republic of Slovenia for the use of animal by‐products C2 (category 2 1069/2009/ ES) for research purposes (permits No. SI B 07‐22‐25, and No. SI B 07‐22‐49, respectively).

#### 2.1.1 Main experiment—mature immunocastrated boars

The experiment was performed in cooperation with a pig breeding company (Besamungsunion Schwein) located in Herzberg, Brandenburg, Germany, between May and July 2018 in two replicates (starting with a 14‐day delay) as only a limited number of sexually mature boars could be provided at one time. For each replicate, 10 sexually mature boars of different breeds and ages (average age: 480 days, minimum 331 days, maximum 1210 days) from an artificial insemination center were used, which had been excluded from further breeding for various reasons (Table S1). The animals were housed in individual pens and fed a commercial feed mixture according to their requirements. IC was performed by a trained veterinarian according to the manufacturer's instructions, with 28‐day intervals between V1 and V2 and between V2 and slaughter (S). The experimental animals were slaughtered in two batches at Westphal Schlachthof GmbH (Herzebrock‐Clarholz, Germany) according to standard procedures (app. 20 h feed deprivation, stunning with CO_2_, and immediate vertical bleeding).

#### 2.1.2 Control experiment—young male pigs

To evaluate the results of the main experiment, we selected a subsample of animals (Pietrain X German Landrace, average age 185 days and weight 120 kg at slaughter) from a larger experiment performed between February and July 2018 at the experimental station of the University of Hohenheim^28^ (Unterer Lindenhof, Eningen, Germany). Six YICs (positive controls) and six EMs (negative controls) were randomly selected for slaughter (Table S2). IC among YICs was performed with a 70‐day interval between V1 and V2 at 84 (V1) and 154 (V2) days of age. The experimental animals were slaughtered in a single batch at 182 days of age (28 days after V2) at the experimental slaughterhouse (Landesanstalt für Schweinezucht Boxberg, Germany) according to standard procedures.

### 2.2 Measurements and sampling procedure

Blood was collected between 9 and 10 a.m. by puncturing the *vena jugularis externa* on the day following V1 (B1), 14 days after V2 (B2), and at exsanguination on the slaughter line (B3) for analysis of hormones and GnRH‐antibody titer. Hot carcass weight (HCW) was recorded at the slaughter line. The reproductive tract was removed, dissected as previously described,^29^ and weighed. The weight of right and left testes, adjacent epididymis, and vesicular and bulbourethral glands (right and left bulbourethral glands, including the urethra; Figure S1) were recorded for each pig. Genital tract, testes, bulbourethral glands, and vesicular glands indices (GTI, TI, BGI, and VGI, respectively) were calculated as the weight of the genital divided by the weight of the warm carcass. Samples of the testes (taken from the area between the tunica albuginea and the mediastinum testis) were collected from the left testis, cut into ∼1 cm^3^ pieces, and fixed in Bouin solution for histological analysis. Additionally, one piece was stored in RNAlater (Sigma–Aldrich, Merck KGaA, Darmstadt, Germany) for mRNA expression analysis. The color of the testicular tissue was assessed on a cross‐section of the left testis, according to the International Commission on Illumination [Commission Internationale de l'éclairage (CIE)] CIE^30^ L* (lightness), a* (redness), and b* (yellowness) color space in triplicate using a Minolta Chroma Meter CR‐300 (Minolta Co., Ltd., Osaka, Japan) with an 11‐mm aperture, D65 illuminant, and calibrated against a white tile. For the determination of boar taint compounds, samples of subcutaneous fat were obtained from the withers, vacuum packed, and stored at −20°C until further laboratory analysis.

### 2.3 Chemical analyses

The total testosterone concentrations in 20 µl plasma were determined using a direct in‐house radioimmunoassay.^28^ Briefly, 20 µl of plasma samples were incubated with [1,2,6,7‐^3^H]‐testosterone (95.5 Ci/mmol; PerkinElmer, Boston, MA, USA) and antiserum (raised against testosterone‐3‐(O‐carboxymethyl)oxime‐bovine serum albumin [3CMO‐BSA] in a rabbit, used at a final dilution of 1:144,000, 67% cross‐reactivity with 5α‐dihydrotestosterone [5αDHT] and <2% for other tested steroids). To compensate for substrate effects, charcoal‐treated plasma (20 µl) was added to the calibration curve. Bound free separation was carried out (using 0.5 ml ice‐cold solution of 0.5% dextran‐coated charcoal in H_2_0) and subsequent centrifugation. Finally, the supernatant was transferred to counting vials with scintillation fluid and counted using a beta counter. For testosterone, the inter‐ and intra‐assay variations were <6% and <9%, respectively. LH (ELISA Genie, London, UK; product number: PRFI00104) and FSH concentrations (Abnova, Taipei, Taiwan; catalog number: KA2342) were determined using porcine‐specific commercial enzyme‐linked immunosorbent assay kits. The concentrations were expressed in nanograms per milliliter of plasma. For LH and FSH concentrations, all samples were analyzed in a single assay, with intra‐assay variations of 12.8 and 12.3%, respectively. The percentage of GnRH binding was measured in the plasma as previously described.^28^ Briefly, GnRH‐iodination was performed using the solid‐phase iodogen method in 1 µg iodogen/cup with 200 µCi^125^I (Na^125^I; Hartmann Analytik GmbH, Braunschweig; I‐RB‐31) and 200 ng GnRH (Fisher Scientific; PEP‐168) diluted in 0.5 M phosphate buffer (pH 7.4). Following 3 min incubation period, the free iodine was separated from the iodinated peptide using an anion‐exchange resin column (specific activity ∼200 nCi/ng GnRH). To determine GnRH binding, 15,000 cpm ^125^I‐GnRH (corresponding to 17.5 pg GnRH) in 100 µl of 0.1 M phosphate buffer were incubated with 5 µl of plasma and 200 µl of 0.1 M phosphate buffer containing 0.1% BSA at 4°C for 24 h. Afterward, bound free separation was carried out with 0.5% dextran‐coated charcoal in 1 ml H_2_0 and subsequent centrifugation. The supernatant was counted for 1 min using a gamma counter. The absolute binding of the biological samples was calculated as counts/total counts. For controls, pooled samples of vaccinated animals with good response (pool+) and non‐vaccinated boars (pool−) were measured within each assay. The specific binding was 39% (range 35–61%) and 4.4% (range 1.3−6.3) for pool+ and pool−, respectively. The inter‐assay variations were <16% for pool+ and <23% for pool−. The androstenone and skatole concentrations were measured in the collected fat samples by high‐performance liquid chromatography (HPLC) as described by Batorek et al.^31^ Briefly, the collected adipose tissue samples were liquefied in a microwave oven for 3 × 1 min at 350 W, transferred to 2.5 ml tubes, and centrifuged for 20 min at 11,200×*g* and 20°C. After centrifugation, the fat was heated to 50°C and 0.5 ± 0.01 g water‐free liquid fat was transferred to 2.5 ml tubes. Next, 1 ml of methanol containing internal standards (0.496 mg/L androstanone and 0.050 mg/L 2‐methylindole) was added to each tube. After stirring for 30 s, the tubes were incubated for 5 min at 30°C in an ultrasonic water bath, placed on ice for 20 min, and centrifuged for 20 min at 11,200×*g* at 4°C. For androstenone determination, 50 µl of the supernatant was subjected to derivatization with dansylhydrazine for exactly 2 min. A 10‐µl aliquot of the derived mixture was then injected into an HPLC column and the fluorescence was detected (excitation at 346 nm and emission at 521 nm) on an HP1200 (Agilent Technologies, Waldbronn, Germany). For skatole determination, 20 µl of the supernatant was injected into the column and fluorescence was detected (excitation at 285 nm and emission at 340 nm) using the same HPLC system. The concentrations were expressed per gram of liquid fat. The detection limits were 0.24 µg/g for androstenone and 0.03 µg/g for skatole. The inter‐ and intra‐assay variations for both compounds were <8 and <10%, respectively.

### 2.4 Testis morphometry

After fixation in Bouin solution, the testis samples were dehydrated and embedded in paraffin (Tissue‐Tek^®^ TEC™ 5 Tissue Embedding Console System; Sakura Finetek Europe HQ, The Netherlands). Next, 5‐µm‐thick sections were processed for routine hematoxylin and eosin (H&E) staining and mounted in Neo‐Mount^®^ (Sigma–Aldrich, Merck KGaA). Histologic images were captured with a Nikon Eclipse Ni‐UM light microscope equipped with a DS‐Fi1 camera and NIS Elements BR 4.6 imaging software (Nikon Instruments Europe B.V., Badhoevedorp, The Netherlands). Composed images were acquired by manually obtaining adjacent image frames and fitting them to a single large image. Representative tissue images were obtained using Adobe Creative Cloud.

To evaluate testicular morphometry, we measured the seminiferous tubule and seminiferous tubule lumen areas on 50–100 randomly selected tubular profiles per animal with circularity >0.95 using a 4× objective lens. The area of the germinal epithelium was calculated for each tubular profile by subtracting the seminiferous tubule lumen area from the seminiferous tubule area. The height of the germinal epithelium was measured at five replicates per tubular profile and then averaged. The Leydig cell morphometry (area of Leydig cells and radius of the nucleus) was evaluated using a 40× objective lens on 100 randomly selected Leydig cells in multiple areas of the interstitium. The nucleus‐to‐cytoplasm (N:C) ratio in Leydig cells was calculated as the nuclear area (calculated from the nuclear radius) divided by the cytoplasmic area. A 63‐intersection point grid was used to assess the volume density of the testicular parenchyma components in 30 adjacent testicular fields. In total, 1890 points were scored for each animal using a 10× objective lens. The points were classified as follows: seminiferous tubule epithelium, seminiferous tubule lumen, Leydig cell, or intertubular compartment (comprising connective tissue, blood, and lymphatic vessels). The volume density (%) was calculated by dividing the number of intersections of the above‐mentioned structures by the total number of intersections.

### 2.5 RNA extraction, cDNA synthesis, and quantitative reverse transcription‐polymerase chain reaction

Total RNA was extracted from testicular tissue samples using RNeasy Mini Kit (Qiagen, Hilden, Germany; catalog number:74104). The 260/280 and 260/230 absorbance ratios were determined using a UV/Vis spectrophotometer (Eppendorf BioSpectrometer^®^; Eppendorf, Hamburg, Germany) to check the purity of the extracted RNA samples. cDNA was synthesized using a High‐Capacity cDNA Reverse Transcription Kit (Thermo Scientific GmbH, Vienna, Austria; catalog number:4368814) according to the manufacturer's instructions. First‐strand cDNA synthesis was performed with RT random primers and reverse transcriptase (Thermo Scientific GmbH) using 1.5 µg of each RNA sample, with 260/280 and 260/230 ratios close to 2.0.

Quantitative polymerase chain reaction (PCR) was performed using a QuantStudio™ 5 Real‐Time PCR System (Applied Biosystems, Thermo Scientific GmbH), with previously synthesized first‐strand cDNA samples as templates. Primers and fluorescent 6‐FAM dye‐labeled minor‐groove‐binder probes/predesigned assays (Table 1) were obtained from Applied Biosystems (Thermo Scientific GmbH). Two endogenous controls—beta‐2‐microglobulin (B‐2‐M) and eukaryotic ribosomal (r) 18s RNA (18s rRNA)—were used for normalization. The quantitative PCRs were performed in a final volume of 10 µl containing 4.5 µl of each reverse transcription sample diluted 100‐fold, 5 µl of TaqMan Universal Master Mix II, and 0.5 µl of TaqMan Gene Expression Assay. As a negative control, quantitative PCR was performed using the same protocol but without cDNA. The PCR efficiency of each primer was tested on pools of EMs (*n* = 12) and IC (*n* = 12); that from the exponential phase was calculated for each amplification plot using the Relative Quantification Analysis Module, version 3.9 (Thermo Fisher Scientific, Applied Biosystems). The average PCR efficiency (Eff) was determined for each plate and used for further calculations. The threshold cycle (Ct) values for B‐2‐M and 18s RNA were used to normalize the Ct values of estrogen receptor 1 (ESR1), estrogen receptor 2, FSH receptor (FSHR), LH/choriogonadotropin receptor, GNRHR‐I, GNRHR‐II, androgen receptor (AR), inhibin subunit beta A, inhibin subunit alpha, hydroxysteroid 17‐beta dehydrogenase 7 (HSD17β7), and steroidogenic acute regulatory protein (STAR) with geometric averaging.^32^ The delta Ct values were calculated using the comparative Ct method^33^ (ΔCt = Ct geometric_mean of controls_ − Ct_target transcript_). A higher ΔCt value indicated higher mRNA expression. Each data point corresponded to three biological replicates. To demonstrate that there was no genomic DNA contamination, control experiments were performed without reverse transcription (RT). The results are presented as median and interquartile range.

**TABLE 1:** List of predesigned TaqMan gene expression assays used for quantitative PCR

Full gene name	Gene	Amplicon length	Assay ID
Estrogen receptor 1	*ESR1*	70	Ss03383398_u1
Estrogen receptor 2	*ESR2*	84	Ss03391479_m1
Follicle‐stimulating hormone receptor	*FSHR*	99	Ss03384581_u1
Luteinizing hormone/choriogonadotropin receptor	*LHCGR*	64	Ss03384991_u1
Gonadotropin‐releasing hormone receptor 1	*GNRHR‐I*	71	Ss03394545_m1
Gonadotropin‐releasing hormone receptor II	*GNRHR‐II*	63	Ss03391559_m1
Androgen receptor	*AR*	86	Ss03822350_s1
Inhibin subunit beta A	*INHIBA*	90	Ss03393536_s1
Inhibin subunit alpha	*INHA*	76	Ss03383260_u1
Hydroxysteroid 17‐beta dehydrogenase 7	*HSD17*β*7*	61	Ss04246893_m1
Steroidogenic acute regulatory protein	*STAR*	73	Ss03381250_u1
Beta‐2‐microglobulin	*B‐2‐M*	60	Ss03391154_m1
Eukaryotic ribosomal (r) 18S rRNA	*18S rRNA*	69	Hs03003631_g1

### 2.6 Statistical analyses

**FIGURE 1:**
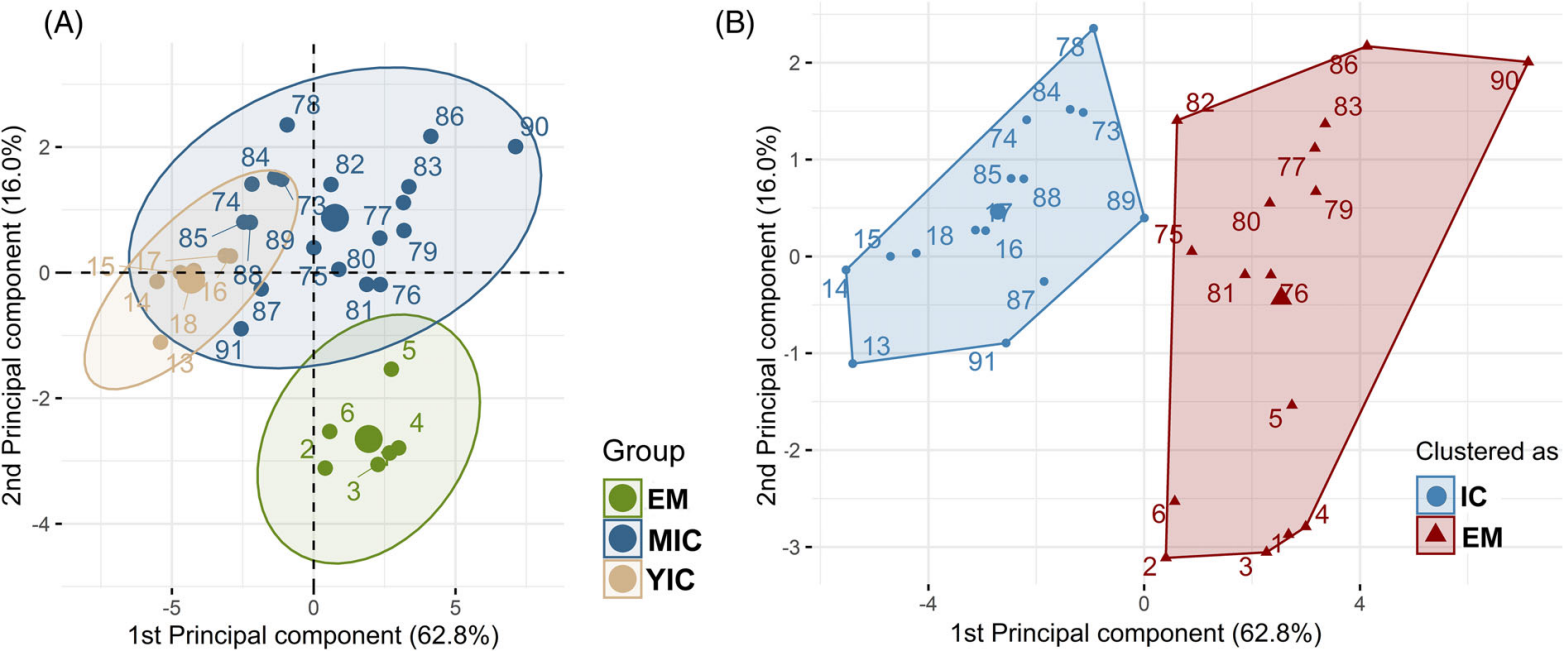
(A) Distribution of boars among all three experimental groups (EMs [green], MICs [blue], and YICs [light brown]) according to the first two principal components of the principal component analysis (PCA) of selected variables related to sexual development and response to immunocastration (ncf. Figure S1). (B) The clusters determined by hierarchical analysis are indicated in different colors (IC: immunocastrated [blue] vs. EMs: entire males [red]). Note that mature immunocastrated boars (MICs) did not appear as a cluster by itself but were grouped either with YICs or with EMs. The cluster centers are marked with larger symbols. Each point (dot or triangle) represents individual pigs (1–6: young entire males [EMs], 13–18: young immunocastrated males [YICs], 73–91: mature immunocastrated boars [MICs])

All statistical analyses were performed using R statistical software (version 3.6.1). Each animal was considered an experimental unit. The detection limits were assigned to animals below or above these limits. Owing to the large differences in the absolute weight of the reproductive organs, body weight, and age between the control and experimental animals (Table S3) and among the experimental animals (range:192–360 kg, 419–2298 g, 484–2982 g, 95–546 g, and 105–1401 g for body weight and weight of the genital tract, testes, bulbourethral glands, and vesicular glands, respectively), the relative weights of the reproductive organs, expressed as an index, were used for statistical analysis. The data for body weight, HCW, redness of testicular tissue (CIE_a), vesicular gland weight, VGI, BGI, TI, androstenone concentration, testosterone concentration, LH concentration, FSH concentration, GnRH binding percentage, testis parenchyma volume density, Leydig cell area, and N:C ratio in Leydig cells were non‐normally distributed and could not be normalized by logarithmic transformation and were not subjected to other transformations. Principal component analysis (PCA) was performed using the R package FactoMiner^34^ and including variables related to sexual development and response to IC, followed by a clustering procedure to evaluate the effect of IC in MICs using the statistical approach described by Parois et al.^35^ Variables with squared cosines >0.5 were selected for PCA and hierarchical clustering on PCA (androstenone, testosterone, and LH concentrations; GnRH antibody binding percentage; GTI, TI, BGI, and VGI; seminiferous tubules, Leydig cells, and germinal epithelium areas; average thickness of the germinal epithelium; Leydig cell nucleus radius; N:C ratio in Leydig cells; and Leydig cell volume density) (Figure S2). Only the first two components of PCA had eigenvalues >1 and together accounted for 78.7% of the inertia. The hierarchical clustering of PCA results of individual animals from all three experimental groups is shown in Figure 1(A), where the three populations (EMs, YICs, and MICs) are distinguished by color. Two clusters (Figure 1(B)) associated with the first two dimensions of PCA were selected based on the shape of the tree and the bar graph of the inertial gain.^34^ MICs did not emerge as a distinct cluster but were grouped with either YICs or EMs; this can also be seen in Figure S3, in which the dendrogram tree has been divided into two parts (clusters).

A non‐parametric statistical model (Wilcoxon rank‐sum test with Bonferroni correction) was used to evaluate the effect of cluster versus positive (YICs) or negative (EMs) controls on the variables related to sexual development and response to IC in MICs and to evaluate the effect of time on the GnRH antibody binding percentage and testosterone concentration in the plasma samples. Statistical significance was set at *p* < 0.05. Data are presented as medians with interquartile ranges.

## 3 RESULTS

In the main experiment, one MIC died (20 days following V2, undetermined cause of death). The remaining 19 MICs, six YICs (positive controls), and six EMs (negative controls) were used for sampling and analysis.

### 3.1 GnRH binding

The GnRH antibody binding increased significantly from V1 to 14 days after V2 (*p* < 0.001; Table 2) in all IC pigs and remained consistent until slaughter (*p* > 0.10; Table 2). However, the MICs that clustered as EMs had significantly lower binding at slaughter than the YIC positive controls and MICs that clustered as immunocastrated (ICs) (*p* < 0.001; Table 2).

**TABLE 2:** Effect of the clusters versus controls on the parameters related to sexual development and response to immunocastration, color of testicular tissue, and histomorphometric parameters (*n* = 31)

		MICs—clustered as^4^		Control		
Parameters^1^, ^2^	MICs^3^—prior clustering (*n* = 19)	IC (*n* = 10)	EM (*n* = 9)	IC (*n* = 6)	EM (*n* = 6)	Cluster effect
Related to sexual development and response to immunocastration						
Androstenone concentration in backfat (µg/g liquid fat)	5.47 [0]	0.71 [4]^ab^	9.08 [3]^c^	0.24 [0]^a^	9.90 [7]^bc^	<0.001
Testosterone concentration at V1 (ng/ml plasma)	3.26 [3]	2.66 [2]^b^	5.09 [4]^b^	0.55 [0]^a^	0.20 [1]^a^	<0.001
Testosterone concentration 2 weeks after V2 (ng/ml plasma)	1.46 [3]	0.52 [0]^b^	3.35 [2]^c^	0.10 [0]^a^	4.58 [13]^c^	<0.001
Testosterone concentration at slaughter (ng/ml plasma)	4.10 [18]	0.80 [1]^b^	19.19 [20]^c^	0.24 [0]^a^	32.34 [9]^c^	<0.001
Luteinizing hormone concentration (ng/ml plasma)	8.12 [10]	4.19 [6]^a^	9.56 [12]^a^	5.21 [1]^a^	29.16 [11]^b^	0.002
Follicle‐stimulating hormone concentration (ng/ml plasma)	0.45 [1]	0.41 [0]^a^	0.53 [1]^b^	0.40 [0]^a^	0.40 [0]^a^	0.019
GnRH antibody binding at V1 (%)	6.08 [0]	6.14 [0]^c^	6.08 [0]^c^	2.87 [0]^b^	2.16 [1]^a^	<0.001
GnRH antibody binding 2 weeks after V2 (%)	36.5 [10]	42.7 [6]^c^	34.1 [6.]^b^	51.6 [8]^c^	2.22 [0]^a^	<0.001
GnRH antibody binding at slaughter (%)	37.6 [10]	45.9 [9]^c^	35.6 [6]^b^	49.9 [8]^c^	1.9 [0]^a^	<0.001
Genital tract index^5^	0.481 [0]	0.337 [0]^a^	0.604 [0]^b^	0.158 [0]^a^	0.581 [0]^b^	<0.001
Testis index^6^	0.504 [0]	0.386 [0]^a^	0.787 [0]^b^	0.308 [0]^a^	0.827 [0]^b^	<0.001
Bulbourethral gland index^7^	0.104 [0]	0.085 [0]^b^	0.127 [0]^c^	0.040 [0]^a^	0.180 [0]^c^	<0.001
Vesicular gland index^8^	0.188 [0]	0.106 [0]^ab^	0.287 [0]^b^	0.021 [0]^a^	0.254 [0]^b^	<0.001
Color of testicular tissue^9^						
CIE L	48.6 [4]	48.6 [6]^b^	48.3 [3]^ab^	53.2 [1]^b^	43.4 [3]^a^	0.001
CIE a	16.8 [6]	11.2 [4]^a^	17.5 [2]^b^	18.0 [1]^ab^	21.3 [2]^c^	<0.001
CIE b	10.2 [2]	10.8 [3]^c^	9.8 [1]^bc^	7.7 [1]^a^	6.8 [2]^a^	<0.001
Testis morphometry^10^						
Seminiferous tubules area (µm^2^)	38741 [4137]	36616 [6265]^b^	41243 [1921]^c^	22314 [5025]^a^	31105 [6595]^b^	<0.001
Germinal epithelium area (µm^2^)	32414 [6906]	27975[6095]^b^	34920 [1386]^c^	18746 [4982]^a^	27139 [6915]^ab^	<0.001
Germinal epithelium height (µm)	59.0 [12]	49.2 [8]^a^	61.2 [4]^b^	43.9 [12]^a^	57.2 [8]^ab^	<0.001
Leydig cell morphometry^11^						
Leydig cell area (µm^2^)	75.7 [60]	53.0 [14]^b^	113.5 [21]^c^	39.6 [7]^a^	128.8 [32]^c^	<0.001
Leydig cell nucleus radius (µm)	2.80 [0]	2.67 [0]^ab^	2.94 [0]^c^	2.42 [0]^a^	2.85 [0]^bc^	<0.001
Nucleus‐to‐cytoplasm ratio in Leydig cells	0.522 [0]	0.726 [0]^a^	0.375 [0]^b^	1.072 [0]^a^	0.271 [0]^b^	<0.001
Testicular parenchyma volume density^12^ (%)						
Seminiferous tubule epithelium	64.0 [8]	63.3 [6]^ab^	64.7 [8]^a^	70.5 [3]^b^	65.9 [4]^ab^	0.014
Seminiferous tubule lumen	9.5 [4]	10.5 [5]	8.6 [4]	8.4 [2]	8.7 [2]	0.518
Leydig cell	11.3 [7]	10.0 [2]^a^	16.4 [7]^b^	8.6 [1]^a^	13.1 [3]^ab^	<0.001
Intertubular compartment	14.4 [3]	15.6 [3]^b^	13.0 [3]^ab^	13.4 [2]^ab^	12.0 [2]^a^	0.016

Values are presented as medians followed by interquartile ranges in brackets.

IC, immunocastrated; EMs, entire males; V1, first vaccination with Improvac® (2 ml, s.c. application, Zoetis); V2, second vaccination with Improvac® (2 ml, subcutaneous application, Zoetis)

* Results (median followed by interquartile ranges in brackets) for the experimental group of mature immunocastrated boars (MICs) without clustering.

^1^ Non‐parametric model (pairwise Wilcox); values are presented as medians followed by interquartile ranges in brackets.

^2^ Medians followed by a different letter differ at *p* < 0.05.

^3^ Data reported for entire experimental group—prior principal component analysis and hierarchical clustering.

^4^ Data reported for two experimental subgroups—obtained by principal component analysis of selected variables related to sexual development and response to immunocastration.

^5^ The genital tract index was calculated as the genital tract weight (weight of the pelvic part of the genital tract, together with the accessory glands and emptied bladder) divided by the warm carcass weight.

^6^ The testis index was calculated as the testis weight (weight of the right and left testes with the epididymis included) divided by the warm carcass weight.

^7^ The bulbourethral gland index was calculated as the bulbourethral gland weight (weight of the right and left bulbourethral glands with the urethra included) divided by the warm carcass weight.

^8^ The vesicular gland index was calculated as the vesicular gland weight divided by the warm carcass weight.

^9^ CIE L, a, b color space; L, higher number denotes a lighter color; a, higher number denotes a redder color; b, higher number denotes a yellower color.

^10^ The testis morphometry was evaluated using a 4× objective lens on 50–100 tubular profiles per animal with circularity above 0.95. Germinal epithelium height was measured at five sites per tubular profile.

^11^ The Leydig cell morphometry was evaluated using a 40× objective lens. A total of 100 Leydig cells were randomly selected. The nucleus‐to‐cytoplasm ratio in Leydig cells was calculated as the cytoplasmic area divided by the nucleus area (calculated from the nucleus radius).

^12^ The testis parenchyma volume densities were evaluated using a 63‐intersection point grid. Thirty adjacent test fields (1890 points) were scored for each animal using a 10× objective lens.

### 3.2 Hormones and boar taint compounds

At V1, MICs had significantly higher testosterone concentrations than the controls (p<0.001, Table 2). A low testosterone concentration at V1 reflects the pre‐pubertal developmental stage of control boars (aged 84 days). Thereafter, we observed a significant increase in testosterone concentrations in EM (negative control) 14 days after V2, which were comparable to those in the MICs that clustered as EMs, whereas testosterone concentrations in YICs and MICs clustered as ICs were significantly lower (*p* < 0.01 and *p* < 0.001; Table 2). At slaughter, the testosterone concentrations in the MICs that clustered as EMs and EM negative controls were very high, which could be related to their response to stress induced by pre‐slaughter procedures,^28^ whereas those in the YICs and MICs that clustered as ICs were significantly lower (*p* < 0.001; Table 2). However, in MICs clustered as ICs, the levels were about 3.5‐fold higher than in YICs (0.80 vs. 0.24 ng/ml plasma), which already exceeded the threshold (0.5 ng/ml) for resumption of testicular function.^36^ Furthermore, the LH concentration in the MICs was similar to those in the YIC controls and lower than in the EM controls (*p* < 0.002; Table 2). The FSH concentrations were low (near the detection limit) in the MICs that clustered as ICs and both controls and slightly elevated in MICs that clustered as EMs (*p* < 0.019; Table 2). Backfat androstenone concentrations were high in the EM controls and MICs that clustered as EMs, intermediate in the MICs that clustered as ICs, and below the threshold (1 ppm) for the risk of boar taint (*p* < 0.001; Table 2).

### 3.3 Reproductive organs

The MICs that clustered as ICs and YIC positive controls showed lower GTI and TI than those in MICs that clustered as EMs and EM negative controls (*p* < 0.001; Table 2). The decrease in TI in the MICs that clustered as ICs was more pronounced than the decrease in GTI (Table 2). The BGI in the MICs that clustered as EMs was similar to those in the EM controls, whereas in the MICs that clustered as ICs was higher than the BGI in YIC controls but lower than in the EM controls (*p* < 0.001; Table 2). The VGI in the MICs that clustered as ICs was intermediate compared with the YIC and EM controls, whereas the VGI in the MICs that clustered as EMs was similar to those in the EM controls (*p* < 0.001; Table 2). The testicular parenchyma of the MICs that clustered as ICs was lighter and less red than the parenchyma in EM controls and more yellow than that of the YIC and EM controls (*p* ≤ 0.001; Table 2). The MICs that clustered as EMs had testicular parenchyma of similar lightness but more yellow than that of the IC and EM controls and less red than that of the EM controls (*p* ≤ 0.001; Table 2). The most pronounced change in the MICs was the lower redness of the testicular parenchyma.

### 3.4 Histological and histomorphometric assessment of testicular tissue

**FIGURE 2:**
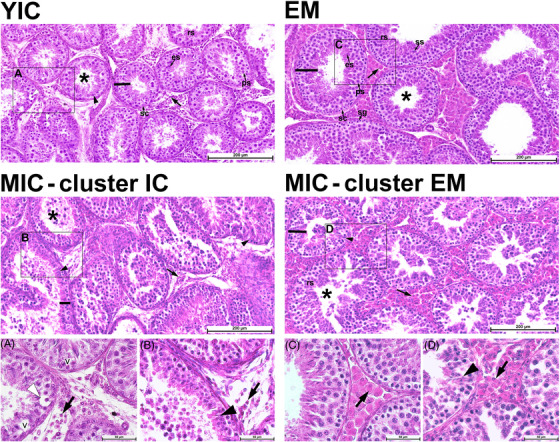
Representative photomicrographs composed of six adjacent image frames of testis cross‐sections showing the seminiferous tubules and interstitial Leydig cells of young (upper row panels) and mature ICs (MICs; middle row panels) boars, as well as higher‐magnification images of the areas marked with black rectangles (lower row panels A–D). Shown are young ICs (YICs; animal No. 14) and entire male (EMs; animal No. 4) boars as positive and negative controls, respectively. MICs (middle row panels) clustered as ICs (MICs—cluster ICs; animal No. 85) or EMs (MICs—cluster EMs; animal No. 77). The lumen of the seminiferous tubule and the germinal epithelium height are marked with an asterisk and a black line, respectively. A drastic reduction in the number and size of Leydig cells (black arrows) is visible in the YICs (positive control; left upper row panel) and the MICs clustered as ICs (left middle row panel) and in higher‐magnification images (*cf*. panels A and B with panels C and D). Exfoliation of germ cells is also seen in all three IC groups (YICs, MICs—cluster ICs, and MICs—cluster EMs). Higher‐magnification images show (A) degeneration of spermatocytes (white arrowheads) and signs of seminiferous epithelium vacuolation (v) and (B, D) spermatid heads retention deep within the seminiferous epithelium (black arrowheads). Hematoxylin and eosin staining, scale bar = 200 and 50 µm; sc, Sertoli cell; ps, primary spermatocytes; ss, secondary spermatocytes; rs, round (spherical) spermatids; es, elongated spermatids.

Representative higher‐magnification photomicrographs of H&E‐stained testis cross‐sections are shown in Figure 2. The derived histomorphometric parameters are summarized in Table 2. The YICs (positive controls) and MICs showed histological signs suggestive of androgen deficiency, including degeneration of spermatocytes and spermatids close to the basal compartment of the seminiferous epithelium, varying degrees of germinal epithelium degeneration with signs of vacuolation and germ cell exfoliation and marked Leydig cell atrophy. Leydig cell atrophy was less evident in the MICs that clustered as EMs, which also maintained some stratification of spermatogenesis (Figure 2).

The results of the histomorphometric analysis (Table 2) provided additional quantitative data that also reflect pronounced change in the Leydig cell size, N:C ratio in Leydig cells and in the volume density of the testicular parenchyma. The Leydig cell area was decreased in MICs that clustered as ICs and was intermediate between the YIC and EM controls; it remained at the same level in the MICs that clustered as EMs and the EM controls (*p* < 0.001; Table 2). The Leydig cell nucleus radius was 1.2‐fold larger in the MICs that clustered as EMs than in the YIC controls and similar to that in the MICs that as ICs and EM controls (*p* < 0.001; Table 2). The N:C ratio in Leydig cells was significantly higher in both the MICs that clustered as ICs and YIC controls compared with the MICs that clustered as EMs and EM negative controls (*p* < 0.001; Table 2). The volume density of the seminiferous tubule epithelium was highest in the YIC controls (70.5%) and lowest in the MICs that clustered as EMs (64.7%), with intermediate density of MICs that clustered as ICs and EM controls (*p* = 0.014; Table 2). The volume density of the seminiferous tubule lumen was constant between the groups and contributed to 8.4–10.5% of the testis parenchyma volume density (*p* = 0.518; Table 2). The intertubular compartment, comprising connective tissue, blood, and lymphatic vessels, was the most expanded among the MICs that clustered as ICs (15.6% of the testicular parenchyma volume density) and the least expanded among the EM controls, with the YIC controls and MICs that clustered as EMs intermediaries (*p* = 0.016; Table 2). The Leydig cell volume density was the most reduced in MICs that clustered as ICs and YIC positive controls (*p* < 0.001; Table 2).

**FIGURE 3:**
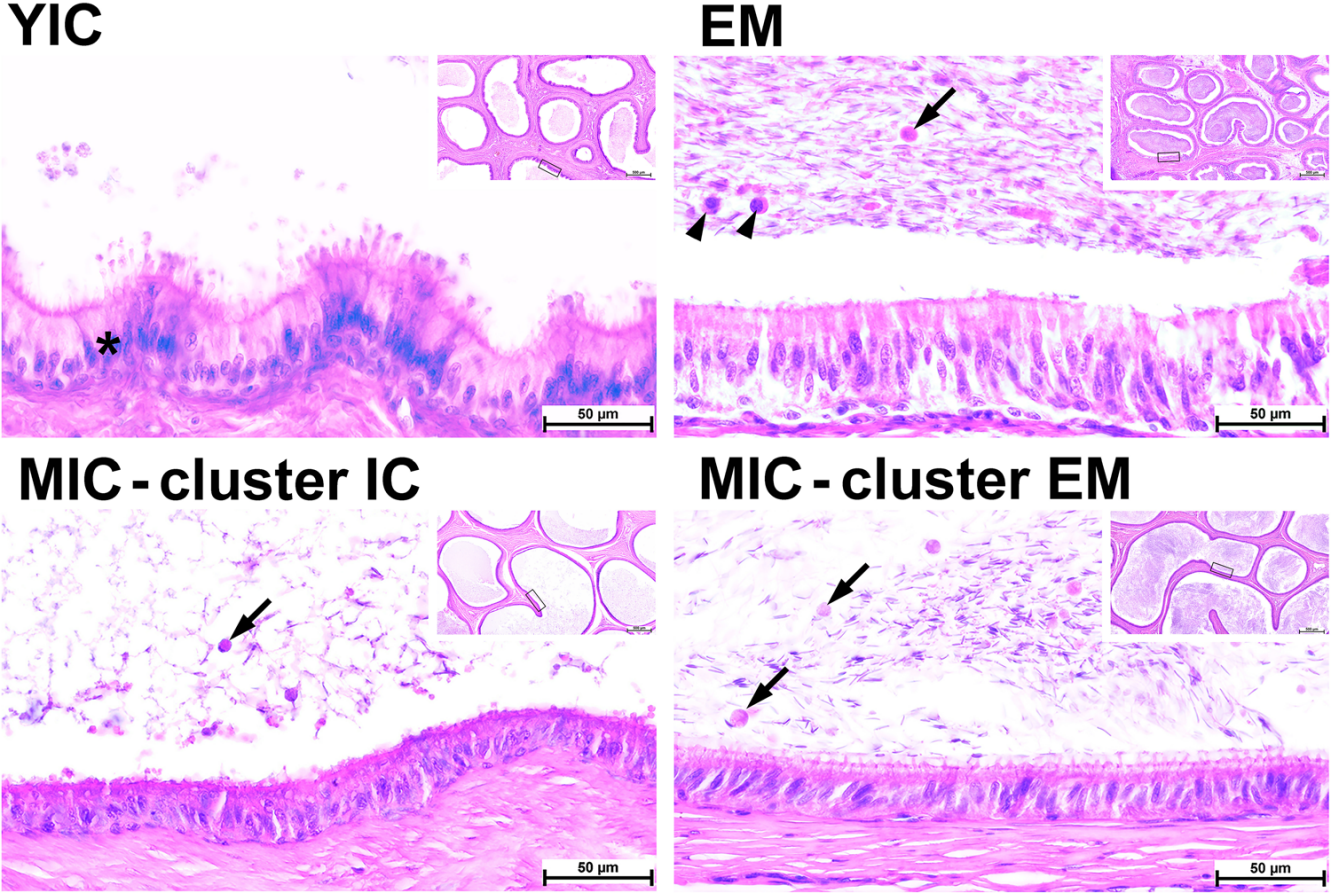
Representative higher‐magnification and overview (inserts) photomicrographs of cauda epididymis cross‐sections. The image shows young ICs (YICs; animal No. 14) and entire male (EMs; animal No. 4) as positive and negative controls, respectively. MICs (lower row panels) clustered as ICs (MICs—cluster ICs; animal No. 85) or EMs (MICs—cluster EMs; animal No. 77). Mature spermatozoa accumulate in the lumen, while cell debris and exfoliated germ cells/spermatids (arrowheads) and round bodies (arrows) are also visible in the lumen. A difference in spermatozoa cell density is visible between the EMs and YICs as well between MICs that clustered as ICs and EMs. Cribriform changes (asterisks) were mainly observed in YICs. Areas shown in higher‐magnification micrographs are marked with rectangles. Hematoxylin and eosin staining, scale bar = 50 and 500 µm (inserts).

We also observed histological alterations in the epididymis (Figure 3). Representative photomicrographs of cauda epididymis cross‐sections are shown in Figure 3. Epididymis duct profiles with sparse spermatozoa cells were more frequent in YICs and MICs that clustered as ICs than in EMs and MICs that clustered as EMs. Profiles with hyperplastic alterations in the epithelium, known as cribriform changes, were mainly observed in the YICs. The luminal contents in both MICs clusters and YICs (exfoliated germ cells/round bodies) were suggestive of indirect changes (i.e., androgen deprivation‐related alteration)^37^; however, they were also observed in EMs. The definitive cause of the cribriform change in the epididymis is not known. However, considering that this was observed in the YICs, it maybe suggestive of pre‐pubertal androgen deprivation.^37^

### 3.5 Testicular mRNA expression

**FIGURE 4:**
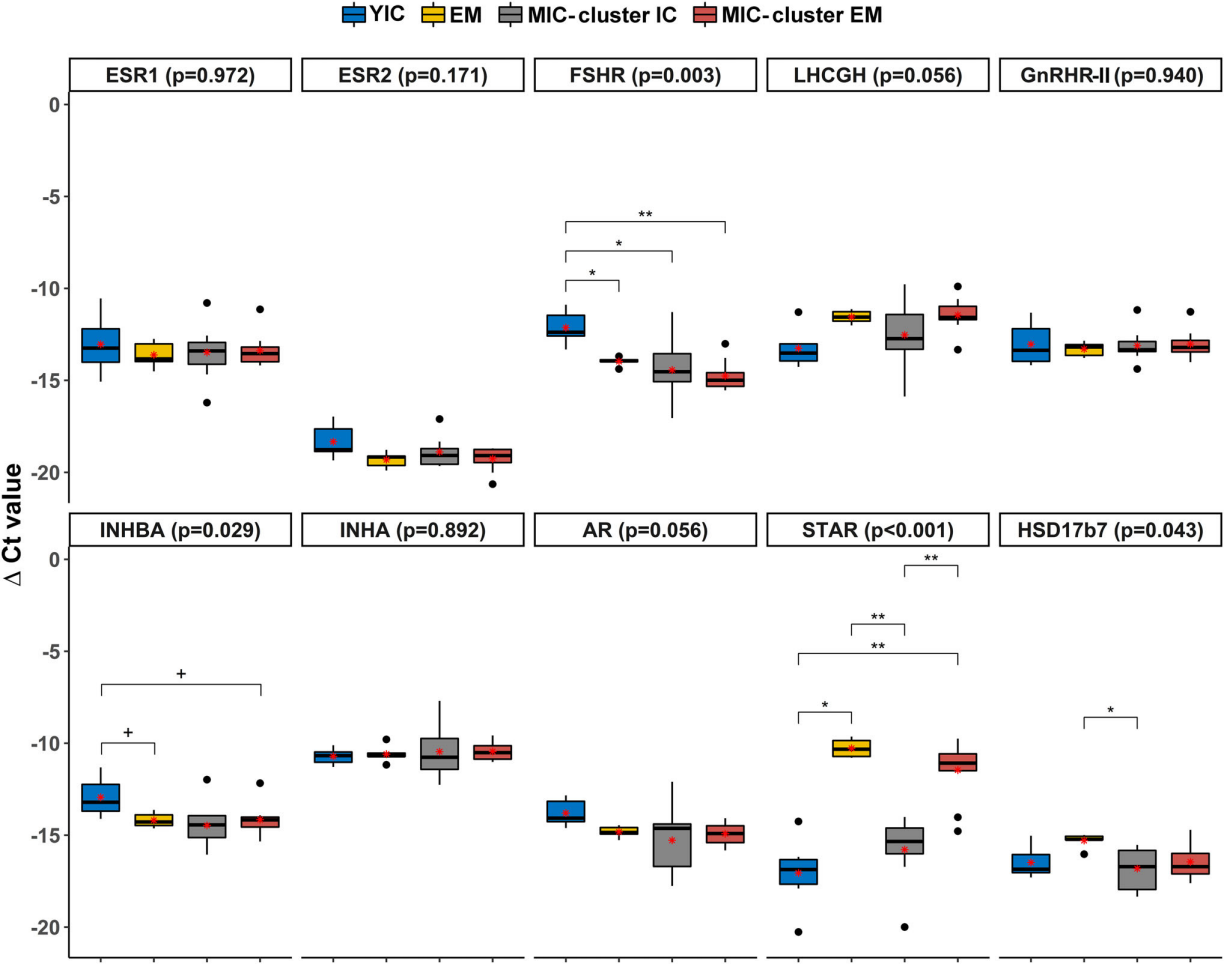
Testicular mRNA expression of ESR1, ESR2, FSHR, LHCGH, GnRHR‐II, INHBA, INHA, AR, STAR, and HSD17β7 in the ICs control group, EMs control group, MICs that clustered as ICs (cluster ICs), and MICs that clustered as EMs (cluster EMs). The ΔCt values are presented as boxplots with medians and interquartile ranges (horizontal lines), means (red star), and total ranges (extreme ends of vertical lines). The levels of significance indicate the effect of clusters within the gene (non‐parametric comparisons): **p<0.01; *p = 0.01–0.05; ^+^ *p* = 0.05–0.10. The ΔCt values were calculated using the comparative Ct method (ΔCt = Ct_geometric mean of controls _−_ _Ct_target transcript_). A higher ΔCt value denotes a higher mRNA expression level. B‐2‐M and 18S rRNA were used as reference genes for the geometric means of the controls. IC, immunocastrated; EM, entire (uncastrated) male; ΔCt, delta cycle threshold; ESR1, estrogen receptor 1; ESR2, estrogen receptor 2; FSHR, follicle‐stimulating hormone receptor; LHCGH, luteinizing hormone/choriogonadotropin receptor; GnRHR2, gonadotropin‐releasing hormone receptor‐II; INHBA, inhibin subunit beta A; INHA, inhibin subunit alpha; AR, androgen receptor; STAR, steroidogenic acute regulatory protein; HSD17β7, hydroxysteroid 17‐beta dehydrogenase 7; B‐2‐M, beta‐2‐microglobulin; 18S rRNA, eukaryotic ribosomal (r) 18S rRNA.

The testicular mRNA expression levels of selected genes related to the control of testicular function and steroidogenesis are presented in Figure 4. Briefly, the expression level of FSHR was significantly higher (*p* < 0.01) in the YICs than in MICs and the EM control. Additionally, the expression levels of INHBA and AR tended to be higher in YICs than in EM controls and MICs that clustered as EMs (*p* < 0.10). The expression level of STAR was lower in the YICs and MICs that clustered as ICs than in the EM controls and MICs that clustered as EMs (*p* < 0.001). Furthermore, the expression level of HSD17β7 was lower in MICs that clustered as ICs than in EM controls (*p* < 0.05). The mRNA expression level of GnRHR‐II was comparable between the groups, whereas the expression level of GnRHR‐I in the testes was below the detection limit.

## 4 DISCUSSION

In recent years, our understanding of the physiological response to androgen deprivation in young boars following IC has increased. Because this method was introduced as an alternative to surgical castration to avoid boar taint (e.g., androstenone and skatole) accumulation in fat tissue, most available data were gathered by applying effective immunization before puberty onset in male pigs of standard slaughter weight (up to 120 kg live weight^18^). IC in young pigs is reported almost fully effective in suppressing sexual maturity^18^, ^38^ up to 154 days after V2.^24^, ^25^, ^39^ During this period, testosterone levels should be below the threshold of 0.5 ng/ml, which would indicate suppression of testicular/Leydig cell function.^36^ However, the timing of the first vaccination in the pre‐ versus post‐pubertal stage of sexual development might play a crucial role in the suppression of reproductive function. Indeed, our results regarding hierarchical clustering following PCA in MICs mature boars as a model to investigate the response to androgen deprivation in sexually mature animals showed a lower response to IC (53%) than normally observed in younger animals receiving V1 in the pre‐pubertal period, usually between 70 and 98 days of age. Although non‐responders are occasionally reported in the literature^26^, ^40^, ^41^ owing to improper vaccination or weak immune response,^42^ probably unrelated to differences in interval length between V1 and V2,^43^ this percentage is considerably lower (0.7, 3, and 4%).^26^, ^40^, ^41^ In our study, all ICs responded immunologically to vaccination, as indicated by the increased GnRH antibody titer and decreased LH serum concentration; however, the response in the MICs that clustered as EMs was lower and not sufficient to either elicit a complete response at the testicular level or reduce/eliminate risk of the boar taint. The genital tract, testis, and accessory sex glands indices, as well as the volume density of Leydig cells in the testis parenchyma and N:C ratio in Leydig cells, which can be used as indicators of endocrine activity, were comparable to those of the EM controls. Accordingly, the plasma testosterone and fat androstenone concentrations remained elevated and were comparable to the values reported for boars in the literature (0.17–50 and 0.53–14.11 ng/ml for testosterone and androstenone, respectively).^44^, ^45^, ^46^ Generally, in YICs, the testosterone and androstenone concentrations in the blood decline rapidly (within 2 weeks) after V2 in response to the lack of LH.^16^, ^47^ However, 14–28 days is needed for the clearance of boar taint compounds from adipose tissue^26^, ^48^, ^49^ as the apparent half‐life of stored androstenone is variable between boars (from 4 to 14 days) and does not depend on the weight of fatty tissue.^50^ Given these findings, animals that show a weaker response to IC at 14 days following V2 (MICs that clustered as EMs) might require an additional booster (V3). In support of the latter, the results of a recent study based only on morphometric measurements of the scrotum, testes, and epididymides suggested that the most suitable protocol for males slaughtered at older ages (heavy pig production) includes three vaccinations starting at approximately 90 days of age.^51^ However, such a vaccination regime cannot be adopted for older boars excluded from breeding centers, in which vaccination starts at a much older age. Bilskis et al.^45^ tested a three‐vaccination protocol to induce IC in mature boars (*n* = 9, average age 1065 days). They observed a significant increase in testosterone concentration in the week following V1, as explained by the lack of feedback from the hypothalamus and a progressive decrease thereafter. The testosterone concentration was positively correlated with boar libido and ejaculate volume and negatively correlated with the percentage of abnormal spermatozoa; conversely, the motility and total sperm number per ejaculate did not correlate with the testosterone concentration. Similarly, Agudelo‐Trujillo et al.^52^ reported that IC effectively reduced boar taint through visual assessment of testis atrophy 35 days after V2 in adult boars (*n* = 12; 885 days of age). Additionally, testosterone levels at slaughter were already elevated in MICs clustered with YICs and exceeded the threshold of 0.5 ng/ml; therefore, it is reasonable to assume that the time for return of testicular/Leydig cell function is likely to be shorter when a two‐dose vaccination regimen is used in older, sexually mature animals. In addition to older age at V1, the shorter interval between V1 and V2 in MICs, that is, 28 days compared with 70 days in YICs, could also contribute to a variable response to IC, although this assumption is speculative and further studies would be needed to clarify this issue.

In contrast, Mitjana et al.^41^ recently reported a variable response to IC in young boars (175 days old) at the morphological level (from no response to complete inhibition of spermatogenesis). Histological studies consistently show moderate to severe reductions in spermatogenesis and a marked reduction in Leydig cell size^24^, ^25^, ^26^, ^41^, ^53^ following IC in young boars, consistent with our findings in mature animals that responded to IC, that is, MICs clustered with YICs. The histological changes in the seminiferous epithelium (seminiferous tubule area, germinal epithelium area, and average germinal epithelium thickness) were unrelated to the GnRH antibody titer and LH concentration but were correlated with testosterone and androsterone concentrations and reproductive tract indices (GTI, TI, BGI, and VGI) in our study. These correlations might differ if the time elapsed between V2 and sampling was longer than the physiological time required for a complete cycle of spermatogenesis in boars (∼40 days).^54^ IC performed in young (pre‐pubertal) pigs suppresses sexual development; when performed in adult (post‐pubertal) pigs, progressive regression of testicular function occurs after lack of GnRH production, as confirmed by our results. The most pronounced change in our and previous studies were the reductions in N:C ratio^41^, ^53^ and Leydig cell size,^26^ followed by a reduction in the number of Leydig cells that were replaced by interstitial tissue, as shown by changes in the testicular parenchyma volume density. While the changes in the seminiferous tubules were less pronounced, they indicated germinal epithelium regression and spermatogenesis disruption. In addition, the CIE color of the testicular parenchyma was lighter and less red in the ICs than in the EMs, suggesting decreased vascularization or blood flow through the testicular tissue during testicular atrophy, as confirmed by a marked decrease in the TI in the ICs and MICs that clustered as ICs compared with that in the controls.

Consistent with our findings in adult boars, men treated for advanced prostate cancer with androgen deprivation therapy (LHRH agonist or antagonist) also showed variable endocrinologic responses, with up to 37.5% of patients failing to achieve basal testosterone concentrations after treatment.^55^ Current androgen deprivation strategies appear to incompletely suppress androgen concentrations and AR‐mediated effects at the tissue level.^56^ Cancer cells are often resistant to androgen deprivation therapy within 1–2 years.^57^ In boars, the GnRH antibody titer gradually declines, and full restoration of testicular function can be expected within 154–280 days after IC.^39^ Considering that in boars, the LH‐independent production of testosterone is attributed to the functional GnRH‐II/GnRHR‐II system in the testes,^8^ the intratesticular increase in GnRHR‐II expression could be responsible for the restoration of reproductive function in IC boars observed previously.^58^ Although the serum testosterone concentration at slaughter was considerably higher in the MICs that clustered as EMs in our study, this theory could not be confirmed by the mRNA expression results. The mRNA expression level of GnRHR‐II was similar in all collected testicular tissue samples, suggesting that some post‐transcriptional mechanisms might interfere with protein production and should also be accounted for. A study in male rats found that a combined immunocontraceptive vaccine consisting of GnRH‐I and ‐II induced significantly higher levels of anti‐GnRH‐I antibodies than either GnRH‐I or GnRH‐II alone.^59^ This observation was quite surprising as laboratory rats are among the mammals in which both the GnRH‐II ligand and GnRHR‐II genes are disrupted (reviewed in Ref. ^6^), and the effect was attributed to the conserved amino acid sequences at the N‐terminus and C‐terminus of GnRH‐I and ‐ II. To our knowledge, such an IC approach has not been tested in pigs that have a functional GnRH2‐GnRHR2 system.

Therefore, future studies should adopt a different approach (e.g., microexcision of Leydig cells for single‐cell quantitative PCR/single‐cell RNA sequencing; combined vaccine) to test the hypothesis regarding the possible involvement of GnRH‐II/GnRHR‐II in IC efficacy/restoration of testicular function after IC. Nevertheless, we identified some novel indications of changes in mRNA expression in testicular tissue among the studied groups. The lower expression levels of STAR, responsible for the transfer of cholesterol in the mitochondria, together with the lower expression level of HSD17β7, responsible for the biosynthesis of steroids from cholesterol, in the YICs and MICs that clustered as ICs, is consistent with the decrease in steroid production and regression of Leydig cells observed after IC. Further, decrease in testosterone levels was observed in the MICs clustered as EMs between first and second sampling (mean values at V1 and 14 days after V2 were 5.09 and 3.35 ng/ml, respectively); however, it was not sufficient to elicit noticeable Leydig cells atrophy. Conversely, the higher expression level of AR observed in YIC controls likely occurred because of a lack of negative feedback from androgens. Similarly, the expression levels of FSHR and INHBA, a pituitary FSH secretion inhibitor, were increased in YIC controls, consistent with the low FSH concentration. Studies on gonadectomized animals demonstrated that FSH promotes biosynthesis and fat accumulation in male and female animals^60^, ^61^, ^62^ through activation of the PPARγ signaling pathway in adipose tissue^60^ and could be responsible for castration‐induced adiposity in boars together with testosterone deficiency. Thus, future studies are needed to assess the expressions levels of FSHR, AR, and ESR1 in adipose tissue of MICs, in addition to mRNA expression linked to Leydig cell endocrinological activity.

## 5 CONCLUSION

Immunocastration in young (pre‐pubertal) pigs effectively suppressed sexual development; however, immunocastration in adult (post‐pubertal) pigs results in progressive regression of testicular function after gonadotropin‐releasing hormone production is reduced. The most pronounced change observed in this study was the reduction in the nuleus‐to‐cytoplasm ratio in Leydig cells; however, the testes index was also a reliable parameter to assess the success rate of immunocastration. For live animals with variable BW and age, endocrinological screening (e.g., assessment of testosterone concentration) should be performed to control the success rate of immunocastration, and a third vaccination should be tested to enhance the immunocastration effect at the testicular level. The mRNA expression levels in testicular tissue linked to Leydig cell endocrinological activity requires further study to explain the lack of endocrinological responses observed in some individuals who responded to immunocastration with lower gonadotropin‐releasing hormone‐antibody titers. Practically speaking, a rapid and reliable measurement method is needed to identify animals on the slaughter line that are weak responders. In our study, the decreased testes index in the matured immunocastrated boars that clustered as immunocastrated was more pronounced than the decreased genital tract index, whereas the color of the testicular parenchyma was lighter and less red in the immunocastrated than in the entire males. However, these observations require confirmation in a larger dataset. Clear cut‐off values should also be established for practical applications.

## CONFLICT OF INTEREST

The authors have nothing to disclose.

## AUTHOR CONTRIBUTIONS

*Conceptualization*: N. B. L., M. Č. P., M. V. *Methodology*: N. B. L., K. K., M. Č. P., G. F., M. Š., K. P., M. V. *Investigation*: N. B. L., K. K., G. F., M. Š., K. P., M. V. *Writing—original draft preparation*: N. B. L. *Writing—review and editing*: N. B. L., K. K., M. Č. P., G. F., M. Š., K. P., R. W., V. S., M. V. *Supervision*: M. Č. P., M. V. *Project administration*: M. Č. P., N. B. L. *Funding acquisition*: M. Č. P., V. S., R. W., N. B. L.

## ETHICS STATEMENT

This study consisted of two experiments. The control experiment was approved by the Ethical Committee for Animal Experiments at the regional level by the authority of Tübingen, Germany (permission for animal experimentation ID HOH 47/17TH). Boars in the main experiment were tested in routine diagnostics conducted by a veterinarian from the Besamungsstation Schwein in Herzberg. According to Directive 2010/63/EU (2010) 119, the study was not subject to ethical protocols.

## Supporting information

Supporting Information

Supporting Information

Supporting Information

Supporting Information

Supporting Information

Supporting Information
