# Supplementary material for: Immunocastration in adult boars as a model for late‐onset hypogonadism
Source: Andrology. 2022 Jul 8;10(6):1217–32. doi: 10.1111/andr.13219 (PMC9545940; doi:10.1111/andr.13219)
Supplement: Supplementary file 1 — Supporting Information [file ANDR-10-1217-s003.docx]

**
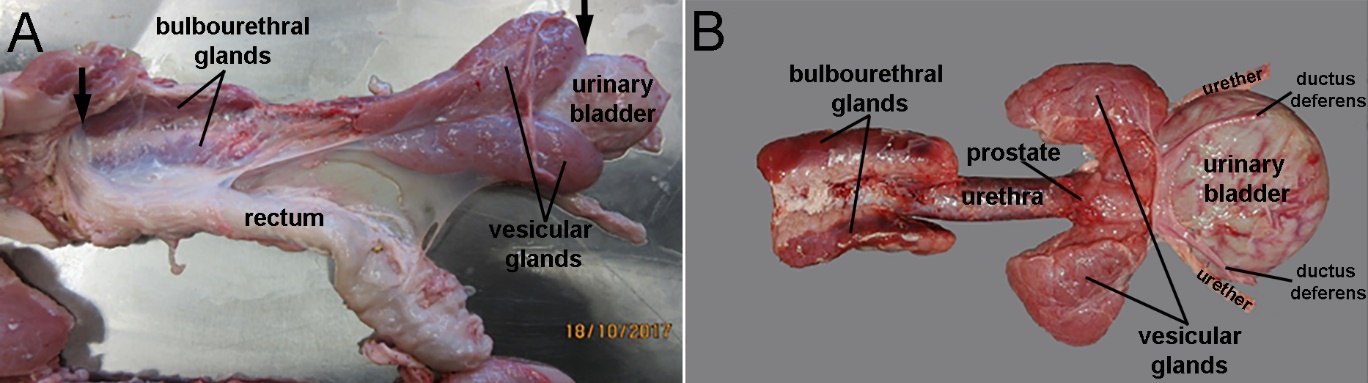
**

**Supplementary Figure 1:** The pelvic segment of the genital tract (in between two ↓), together with prostate, bulbourethral and vesicular glands and the emptied bladder in intact (A) and dissected (B) form.
