# Supplementary material for: Immunocastration in adult boars as a model for late‐onset hypogonadism
Source: Andrology. 2022 Jul 8;10(6):1217–32. doi: 10.1111/andr.13219 (PMC9545940; doi:10.1111/andr.13219)
Supplement: Supplementary file 2 — Supporting Information [file ANDR-10-1217-s006.docx]

**
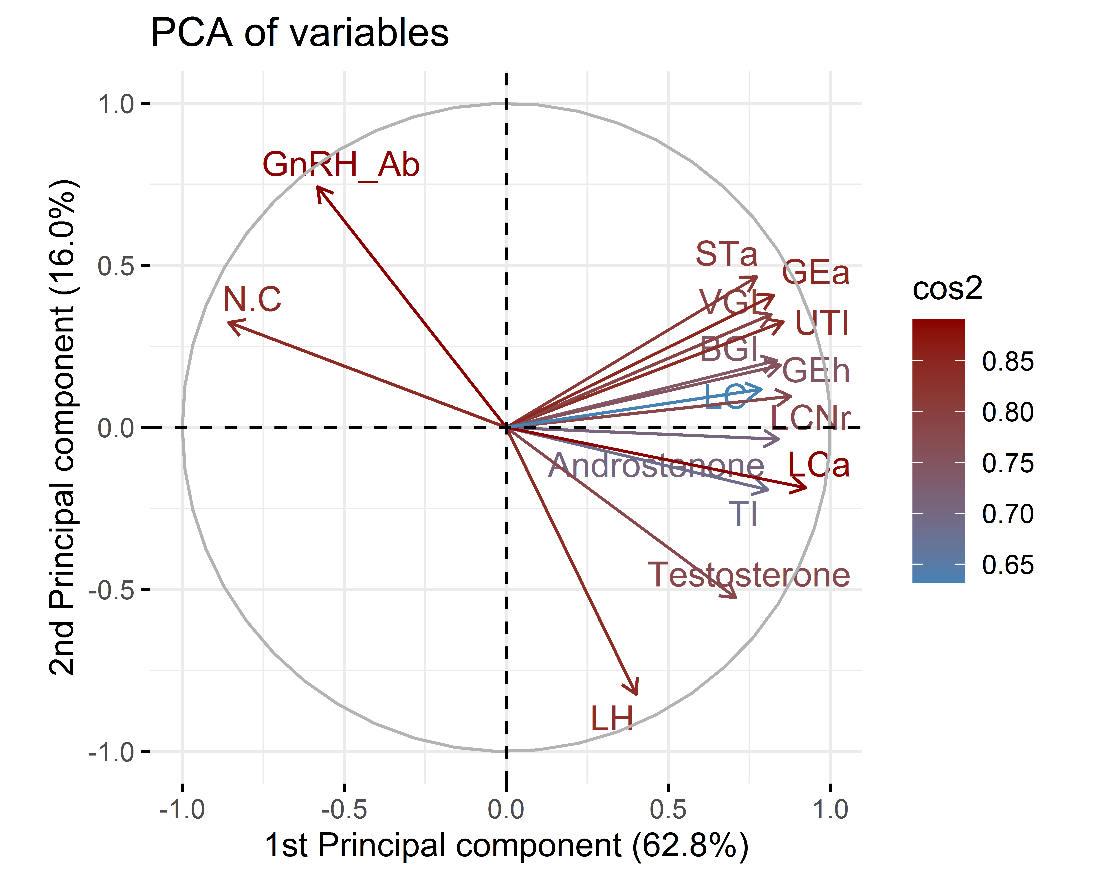
**

**Supplementary Figure 2:** Principal component analysis (n=31) representing the relationships between selected variables (n=15) related to sexual development and response to immunocastration. The biplot of the first two principal components jointly represents 78.7% of the total variation. Androstenone, androstenone concentration in backfat (µg/g liquid fat); Testosterone, testosterone concentration at slaughter (ng/mL plasma); GnRH_Ab, gonadotropin-releasing hormone antibody binding at slaughter (%); LH, luteinizing hormone concentration at slaughter (ng/mL plasma); GTI, genital tract index, calculated as the genital tract weight (weight of the pelvic part of the genital tract, accessory glands, and emptied bladder) divided by the warm carcass weight; TI, testis index, calculated as the testis weight (weight of the right and left testes and epididymis included) divided by the warm carcass weight; BGI, bulbourethral gland index, calculated as the bulbourethral gland weight (weight of the right and left bulbourethral glands and urethra) divided by the warm carcass weight; VGI, vesicular gland index, calculated as the vesicular gland weight divided by the warm carcass weight; STa, seminiferous tubules area (µm^2^); GEa, germinal epithelium area (µm^2^); GEh, average germinal epithelium thickness (µm); LCa, Leydig cell area (µm^2^); LCNr, Leydig cell nucleus radius (µm); N:C ratio, nucleus-to-cytoplasm ratio in Leydig cells, calculated as the cytoplasm area divided by the nucleus area (calculated from the nucleus radius); LC, Leydig cell percentage in the testis parenchyma; cos2, squared cosine.
