# Supplementary material for: Immunocastration in adult boars as a model for late‐onset hypogonadism
Source: Andrology. 2022 Jul 8;10(6):1217–32. doi: 10.1111/andr.13219 (PMC9545940; doi:10.1111/andr.13219)
Supplement: Supplementary file 3 — Supporting Information [file ANDR-10-1217-s001.docx]

**
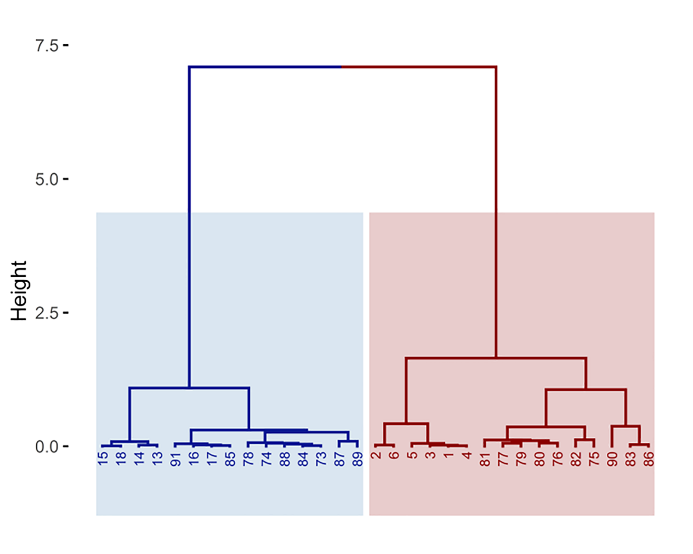
**

**Supplementary Figure 3:** The cluster dendrogram of individual pigs [1–6: young entire males (EM), 13–18: young immunocastrated males (YIC), 73–91: mature immunocastrated boars (MIC)]. Note that cluster dendrogram tree is divided into two parts (clusters)- blue, 13–18: YIC and part of mature immunocastrated boars (MIC) clustered with YIC; and a red part, 1–6: young entire males (EM) and part of MIC clustered with EM. Within EM (red cluster), separation between EM and MIC clustered with EM can be observed, whereas this is not the case for immunocastrated animals (blue cluster).
