# Supplementary material for: Immunocastration in adult boars as a model for late‐onset hypogonadism
Source: Andrology. 2022 Jul 8;10(6):1217–32. doi: 10.1111/andr.13219 (PMC9545940; doi:10.1111/andr.13219)
Supplement: Supplementary file 4 — Supporting Information [file ANDR-10-1217-s004.docx]

**Supplementary Table 1.** Data on the experimental animals (mature immunocastrated boars)

| **Animal ID** | **Age at slaughter (days)** | **Warm carcass weight (kg)** | **Breed** | **Reason for removal** |
| --- | --- | --- | --- | --- |
| **73** | 1210 | 278 | Pietrain | End of trial |
| **74** | 333 | 159 | Duroc | Azoospermia |
| **75** | 331 | 169 | Duroc | Denier |
| **76** | 331 | 156 | Duroc | Denier |
| **77** | 538 | 258 | Duroc | Denier |
| **78** | 572 | 220 | Duroc | Denier |
| **79** | 435 | 193 | Duroc | Low sperm quality |
| **80** | 438 | 184 | Duroc | Low sperm quality |
| **81** | 588 | 261 | Duroc | Denier |
| **82** | 424 | 197 | Duroc | Denier |
| **83** | 644 | 282 | Duroc | Denier |
| **84** | 766 | 288 | Duroc | Denier |
| **85** | 345 | 153 | Duroc | Denier |
| **86** | 420 | 227 | Duroc | Low sperm quality |
| **87** | 480 | 200 | Duroc | Low sperm quality |
| **88** | 548 | 218 | Duroc | Low sperm quality |
| **89** | 685 | 250 | Duroc | Denier |
| **90** | 595 | 213 | Large White | Low sperm quality |
| **91** | 428 | 236 | Duroc | Low sperm quality |
