# Supplementary material for: Immunocastration in adult boars as a model for late‐onset hypogonadism
Source: Andrology. 2022 Jul 8;10(6):1217–32. doi: 10.1111/andr.13219 (PMC9545940; doi:10.1111/andr.13219)
Supplement: Supplementary file 5 — Supporting Information [file ANDR-10-1217-s005.docx]

**Supplementary Table 2.** Data on control animals (young male immunocastrated pigs and young entire males as positive and negative controls, respectively)

| **Animal ID** | **Treatment/sex** | **Age at slaughter (days)** | **Warm carcass weight (kg)** | **Breed** |
| --- | --- | --- | --- | --- |
| **1** | entire male | 179 | 94.0 | Pietrain X German Landrace |
| **2** | entire male | 185 | 88.9 | Pietrain X German Landrace |
| **3** | entire male | 177 | 79.9 | Pietrain X German Landrace |
| **4** | entire male | 186 | 112.3 | Pietrain X German Landrace |
| **5** | entire male | 180 | 87.2 | Pietrain X German Landrace |
| **6** | entire male | 185 | 89.4 | Pietrain X German Landrace |
| **13** | immunocastrate | 188 | 97.7 | Pietrain X German Landrace |
| **14** | immunocastrate | 185 | 99.3 | Pietrain X German Landrace |
| **15** | immunocastrate | 187 | 97.8 | Pietrain X German Landrace |
| **16** | immunocastrate | 186 | 100.0 | Pietrain X German Landrace |
| **17** | immunocastrate | 179 | 103.6 | Pietrain X German Landrace |
| **18** | immunocastrate | 185 | 89.6 | Pietrain X German Landrace |
