# Supplementary material for: Immunocastration in adult boars as a model for late‐onset hypogonadism
Source: Andrology. 2022 Jul 8;10(6):1217–32. doi: 10.1111/andr.13219 (PMC9545940; doi:10.1111/andr.13219)
Supplement: Supplementary file 6 — Supporting Information [file ANDR-10-1217-s002.docx]

**Supplementary Table 3.** Biometric data of the experimental (mature immunocastrated (IC) boars - prior, and after clustering) and control animals (young immunocastrates (YIC) and entire males (EM) as positive controls and negative controls, respectively). Values are reported as medians followed by interquartile ranges in parentheses.

| Parameters | **Mature IC boars – prior clustering^1^ (n=19)** | **Mature IC boars – clustered as^2^** | | **Controls** | |
| --- | --- | --- | --- | --- | --- |
|  |  | **IC (n=10)** | **EM (n=9)** | **YIC (n=6)** | **EM (n=6)** |
| Age at slaughter (days) | 480 [170] | 548 [257] | 437 [155] | 186 [2] | 183 [6] |
| Body weight at slaughter (kg) | 273 [82] | 275 [63] | 256 [81] | 124 [3] | 112 [6] |
| Warm carcass weight (kg) | 218 [66] | 220 [50] | 205 [65] | 99 [2] | 89 [5] |
| Genital tract weight^3^ (g) | 997 [414] | 918 [356] | 1233 [851] | 152 [64] | 625 [217] |
| Testis weight^4^ (g) | 1148 [505] | 910 [391] | 1421 [550] | 302 [78] | 668 [192] |
| Bulbourethral glands weight^5^ (g) | 228 [161] | 207 [85] | 331 [254] | 39 [15] | 164 [54] |
| Vesicular gland weight (g) | 439 [311] | 317 [305] | 582 [562] | 21 [14] | 303 [138] |

^1^ Data reported for entire experimental group – prior principal component analysis and hierarchical clustering.

^2^ Data reported for two experimental subgroups – obtained by principal component analysis of selected variables related to sexual development and response to immunocastration.

^3^Genital tract weight = weight of the pelvic part of the genital tract together with the accessory glands and emptied bladder

^4^Testis weight = weight of the right and left testes (epididymis included)

^5^Bulbourethral gland weight = weight of the right and left bulbourethral glands (urethra included)
